# Association of Ambient Fine Particulate Matter Air Pollution With Kidney Transplant Outcomes

**DOI:** 10.1001/jamanetworkopen.2021.28190

**Published:** 2021-10-07

**Authors:** Su-Hsin Chang, Massini Merzkani, Haris Murad, Mei Wang, Benjamin Bowe, Krista L. Lentine, Ziyad Al-Aly, Tarek Alhamad

**Affiliations:** 1 Division of Public Health Sciences, Department of Surgery, Washington University School of Medicine in St. Louis, Missouri; 2 Institute for Public Health, Washington University School of Medicine in St. Louis, Missouri; 3 Division of Nephrology, Washington University School of Medicine in St. Louis, Missouri; 4 Transplant Epidemiology Research Collaboration (TERC), Institute for Public Health, Washington University School of Medicine in St. Louis, Missouri; 5 Clinical Epidemiology Center, Research and Education Service, VA St. Louis Health Care System, St. Louis, Missouri; 6 Center for Abdominal Transplantation, Saint Louis University School of Medicine, St. Louis, Missouri

## Abstract

**Importance:**

Increased levels of ambient fine particulate matter (PM_2.5_) air pollution are associated with increased risks for detrimental health outcomes, but risks for patients with kidney transplants (KTs) remain unknown.

**Objective:**

To investigate the association of PM_2.5_ exposure with KT outcomes.

**Design, Setting, and Participants:**

This retrospective cohort study was conducted using data on patients who received KTs from 2004 to 2016 who were identified in the national US transplant registry and followed up through March 2021. Multiple databases were linked to obtain data on PM_2.5_ concentration, KT outcomes, and patient clinical, transplant, and contextual factors. Data were analyzed from April 2020 through July 2021.

**Exposures:**

Exposures included post-KT time-dependent annual mean PM_2.5_ level (in 10 μg/m^3^) and mean PM_2.5_ level in the year before KT (ie, baseline levels) in quartiles, as well as baseline annual mean PM_2.5_ level (in 10 μg/m^3^).

**Main Outcomes and Measures:**

Acute kidney rejection (ie, rejection within 1 year after KT), time to death-censored graft failure, and time to all-cause death. Multivariable logistic regression for kidney rejection and Cox analyses with nonlinear assessment of exposure-response for death-censored graft failure and all-cause death were performed. The national burden of graft failure associated with PM_2.5_ levels greater than the Environmental Protection Agency recommended level of 12 μg/m^3^ was estimated.

**Results:**

Among 112 098 patients with KTs, 70 522 individuals (62.9%) were older than age 50 years at the time of KT, 68 117 (60.8%) were men, and the median (IQR) follow-up was 6.0 (3.9-8.9) years. There were 37 265 Black patients (33.2%), 17 047 Hispanic patients (15.2%), 48 581 White patients [43.3%]), and 9205 patients (8.2%) of other race or ethnicity. The median (IQR) baseline PM_2.5_ level was 9.8 (8.3-11.9) μg/m^3^. Increased baseline PM_2.5_ level, compared with quartile 1 baseline PM_2.5_ level, was not associated with higher odds of acute kidney rejection for quartile 2 (adjusted odds ratio [aOR], 0.99; 95% CI, 0.92-1.06) but was associated with increased odds for quartile 3 (aOR, 1.11; 95% CI, 1.04-1.20) and quartile 4 (aOR, 1.13; 95% CI, 1.05-1.23). Nonlinear assessment of exposure-response for graft failure and death showed no evidence for nonlinearity. Increased PM_2.5_ levels were associated with increased risk of death-censored graft failure (adjusted hazard ratio [aHR] per 10 μg/m^3^ increase, 1.17; 95% CI, 1.09-1.25) and all-cause death (aHR per 10 μg/m^3 ^increase, 1.21; 95% CI, 1.14-1.28). The national burden of death-censored graft failure associated with PM_2.5_ above 12 μg/m^3^ was 57 failures (95% uncertainty interval, 48-67 failures) per year among patients with KTs.

**Conclusions and Relevance:**

This cohort study found that PM_2.5_ level was an independent risk factor associated with acute rejection, graft failure, and death among patients with KTs. These findings suggest that efforts toward decreasing levels of PM_2.5_ concentration may be associated with improved outcomes after KT.

## Key Points

**Question:**

Is exposure to increased levels of ambient fine particulate matter (PM_2.5_) air pollution associated with increased risk of adverse posttransplant outcomes among patients with kidney transplants?

**Findings:**

In this cohort study of 112 098 patients with kidney transplants, increased PM_2.5_ concentration was associated with increased risk of adverse posttransplant outcomes, including acute rejection, graft failure, and death.

**Meaning:**

These findings suggest that health outcomes associated with air pollution may extend to serious adverse clinical outcomes among patients with kidney transplants.

## Teaser

This cohort study investigates the association of exposure to ambient fine particulate matter air pollution with kidney transplant outcomes.

## Introduction

Increased levels of ambient air pollution (ie, fine particulate matter 2.5 μm or less in aerodynamic diameter [PM_2.5_]) are associated with an increased risk of detrimental health outcomes, including cardiovascular disease, diabetes, and all-cause mortality.^1,2,3,4,5,6^ The underlying mechanisms for these associations may include associations of inhaled particulate matter with increased sympathetic vascular modulation, intravascular thrombosis, and promotion of atherosclerosis.^7,8^ A dose-response association has also been reported.^9^ Furthermore, Dockery et al^10^ found that improvements in air quality, with decreases in PM_2.5_ levels, were associated with a decrease in mortality risk.

In the field of kidney disease, epidemiological studies from 2016 to 2020^11,12,13^ have found that increased levels of PM_2.5_ are associated with increased risk for decline in kidney functions, including decreased estimated glomerular filtration rate (eGFR) and increased rates of chronic kidney disease (CKD) and end-stage kidney disease. The etiology of kidney disease may be mediated by an increase in systemic inflammation and oxidative stress associated with air pollutants.^14,15^ It also has been found that particulate matter inhaled through the respiratory tract and cleared by the kidney may be associated with direct damage to renal tissue.^16^ Furthermore, air pollutants and PM_2.5_ are associated with insulin resistance,^17,18^ attenuated flow-mediated arterial dilation,^19^ and systemic hypertension,^20,21,22^ which are important factors that may be associated with kidney function.

Despite the existing evidence for an association between PM_2.5_ levels and health outcomes, few studies have examined the association between PM_2.5_ levels and the outcomes of solid organ transplantation. Among patients with lung transplants, Bhinder et al^23^ found that increased PM_2.5_ levels were associated with an increased risk of chronic lung allograft dysfunction and overall mortality. Similar findings have been observed among individuals with heart transplants.^24^ Among individuals with kidney transplant (KT), studies^25,26^ found that exposure to air pollutants was associated with an increased risk of cardiovascular mortality, but associations of PM_2.5_ levels with other important transplant outcomes have not been examined to date, to our knowledge. With the identified knowledge gap in the association of PM_2.5_ levels with KT outcomes, this study aimed to determine whether PM_2.5_ concentration is an independent risk factor associated with kidney rejection, graft failure, or overall mortality among patients with KTs.

## Methods

Exemptions for study approval and informed consent were obtained for this cohort study from the Washington University in St. Louis School of Medicine Institutional Review Board because the study was secondary analyses of deidentified data. This report follows the Strengthening the Reporting of Observational Studies in Epidemiology (STROBE) reporting guideline for cohort studies.

### Study Design and Data

A retrospective cohort of patients who received KTs from 2004 to 2016 was obtained from the Organ Procurement and Transplantation Network (OPTN). Transplant outcomes were followed up until March 2021. Detailed descriptions of OPTN data were described elsewhere.^27,28^ Briefly, the database contains national data on the candidate waiting list, organ donation and matching, and transplantation.^29^

We obtained recipient characteristics, including age, sex, race and ethnicity (as reported by transplant centers in electronic health records), body mass index (BMI; calculated as weight in kilograms divided by height in meters squared), insurance status, and zip code of residence at the time of KT, as well as clinical data on panel reactive antibodies (PRA), diabetes, chronic obstructive pulmonary disease, etiology of kidney disease, and duration of dialysis closest to transplant. Race and ethnicity were among the many recipient and donor characteristics (eg, age and sex) that we used to adjust for recipient and donor characteristics. We also collected donor characteristics, including age, sex, race and ethnicity, BMI, kidney donor profile index, donor type, and history of hypertension, as well as transplant factors, including donor-recipient cytomegalovirus seropairing, level of human leukocyte antigen (HLA) mismatch, and cold ischemia time. Patient zip codes of residence were mapped to zip code–level data to obtain contextual characteristics, including area deprivation index (ADI), population density (measured as number of individuals per square meter), median income, high school graduation and unemployment rates, and proportion of residents below the federal poverty line. Data on ADI were obtained from the University of Wisconsin School of Medicine and Public Health’s Neighborhood Atlas.^30,31^ ADI summarizes factors for the theoretical domains of income, education, employment, and housing quality.^32^ Data on population density were obtained from the US Bureau of Census 2010 Zip Code Tabulation Area to tract Relationship File.^33^ Data on remaining contextual characteristics were obtained from 2011 to 2015 American Community Survey 5-year estimates accessed through ArcGIS Living Atlas of the World.^34^

### Exposure

The exposure was post-KT time-dependent annual mean PM_2.5_ concentration in micrograms per cubic meter in the recipient residential zip code area to reflect changing levels of PM_2.5_ over time after KT. Data on annual mean PM_2.5_ concentration (1 × 1 km) in the contiguous United States were obtained from aerosol optical depth retrievals from the NASA Moderate Resolution Imaging Spectroradiometer, Multi-Angle Imaging Spectroradiometer, and Sea-Viewing Wide Field-of-View Sensor calibrated using geographically weighted regression.^35,36^ The years of availability were 2001 to 2018. The overlap of 1 × 1–km resolution PM_2.5_ grids and zip code area produced surface area–weighted PM_2.5_ levels for each zip code.^11^

Baseline PM_2.5 _level, defined as the annual mean PM_2.5_ level in the year before KT for a patient’s residential zip code area, was also considered as baseline PM_2.5_ level in 10 μg/m^3^ increments and baseline PM_2.5_ level categorized in quartiles.

### Outcomes

The outcomes included acute kidney rejection reported at 1 year after KT (yes or no), death-censored graft failure (yes or no; if yes, time from KT to graft failure), and all-cause death (yes or no; if yes, time from KT to patient death), with all data from the OPTN. The latter 2 transplant outcomes were followed up until March 2021 unless otherwise specified.

### Analytic Cohort

The cohort included patients who underwent KT from 2004 to 2016. We excluded patients aged younger than 18 years at KT, patients receiving kidneys from living donors, patients with a previous KT, and patients with missing data on zip codes or continuous covariate variables.

### Statistical Analysis

Recipient characteristics, donor characteristics, transplant factors, and contextual factors were stratified by baseline PM_2.5_ level quartiles in percentages for categorical variables and in medians (IQRs) for continuous variables. To compare between baseline PM_2.5_ level quartiles, χ^2^ tests were performed for categorical variables and Kruskal-Wallis tests were performed for continuous variables. Survival curves for time-to-event transplant outcomes (ie, death-censored graft failure and all-cause death) stratified by baseline PM_2.5_ level quartiles were adjusted for recipients' age, sex, race and ethnicity, dialysis status and duration, and ADI.

The associations of post-KT PM_2.5_ concentration with time-to-event transplant outcomes were analyzed by multivariable Cox models. Robust sandwich variance estimators for Cox models were used.^37,38^ Follow-up was censored at the end of 2018 because data on annual mean PM_2.5_ concentration were available only up to 2018. The covariates in these multivariable models were selected from all recipient demographics, donor characteristics, transplant factors, contextual factors (see Table for included variables), and year of KT using the forward-selection algorithm with a stopping rule informed by Akaike information criterion (smallest). Year of KT was included in all analyses to account for decreasing annual mean PM_2.5_ concentration over time. The same analyses were repeated for the association of baseline PM_2.5_ exposures with time-to-event transplant outcomes. Association of baseline PM_2.5_ level quartiles with the binary transplant outcome (ie, acute kidney rejection) was analyzed using multivariable logistic regression.

**Table.** Recipient and Donor Characteristics and Transplant and Contextual Factors by Quartile

Variable	Overall (N = 112 098)	Patients by PM_2.5_ concentration quartile, No. (%)^a^				*P* value
		Quartile 1 (n = 28 025)^b^	Quartile 2 (n = 28 024 )^c^	Quartile 3 (n = 28 025)^d^	Quartile 4 (n = 28 024)^e^	
**Recipient characteristic**						
Age, y						
18-50	41 576 (37.09)	9612 (34.30)	10 360 (36.97)	10 622 (37.90)	10 982 (39.19)	<.001
>50	70 522 (62.91)	18 413 (65.70)	17 664 (63.03)	17 403 (62.10)	17 042 (60.81)	
Sex						
Women	43 981 (39.23)	10 956 (39.09)	11 175 (39.88)	10 968 (39.14)	10 882 (38.83)	.07
Men	68 117 (60.77)	17 069 (60.91)	16 849 (60.12)	17 057 (60.86)	17 142 (61.17)	
Race and ethnicity						
Black	37 265 (33.24)	5148 (18.37)	9572 (34.16)	11 202 (39.97)	11 343 (40.48)	<.001
Hispanic	17 047 (15.21)	4323 (15.43)	4130 (14.74)	3537 (12.62)	5057 (18.05)	
White	48 581 (43.34)	15 765 (56.25)	12 099 (43.17)	11 255 (40.16)	9462 (33.76)	
Other^f^	9205 (8.21)	2789 (9.95)	2223 (7.93)	2031 (7.25)	2162 (7.71)	
BMI						
<18.5	38 083 (1.82)	9555 (1.66)	9459 (1.78)	9453 (1.72)	9616 (2.13)	<.001
18.5 to <25	2043 (28.52)	465 (27.8)	498 (27.18)	482 (28.12)	598 (30.97)	
25 to <30	31 967 (33.97)	7790 (34.09)	7618 (33.75)	7881 (33.73)	8678 (34.31)	
≥30	39 684 (35.4)	10 192 (36.37)	10 423 (37.19)	10 138 (36.17)	8931 (31.87)	
Missing	321 (0.29)	23 (0.08)	26 (0.09)	71 (0.25)	201 (0.72)	
Insurance						
Public	82 763 (73.83)	20 870 (74.47)	21 050 (75.11)	20 531 (73.26)	20 312 (72.48)	<.001
Private	29 197 (26.05)	7127 (25.43)	6948 (24.79)	7460 (26.62)	7662 (27.34)	
Other	138 (0.12)	28 (0.1)	26 (0.09)	34 (0.12)	50 (0.18)	
PRA, %						
0	67 298 (60.03)	17 668 (63.04)	16 986 (60.61)	16 746 (59.75)	15 898 (56.73)	<.001
>0 to 20	17 170 (15.32)	3516 (12.55)	3776 (13.47)	4361 (15.56)	5517 (19.69)	
>20-80	16 202 (14.45)	4093 (14.60)	4298 (15.34)	4088 (14.59)	3723 (13.29)	
>80	10 063 (8.98)	2594 (9.26)	2808 (10.02)	2555 (9.12)	2106 (7.51)	
Missing	1365 (1.22)	154 (0.55)	156 (0.56)	275 (0.98)	780 (2.78)	
Dialysis time, mo						
0	13 137 (11.72)	3543 (12.64)	3204 (11.43)	3251 (11.6)	3139 (11.20)	<.001
<24	15 708 (34.15)	4117 (35.38)	3781 (34.61)	3895 (34.36)	3915 (32.24)	
24-60	38 281 (14.01)	9915 (14.69)	9700 (13.49)	9630 (13.90)	9036 (13.97)	
>60	31 544 (28.14)	7272 (25.95)	8211 (29.3)	7691 (27.44)	8370 (29.87)	
Missing	13 428 (11.98)	3178 (11.34)	3128 (11.16)	3558 (12.70)	3564 (12.72)	
Etiology of kidney disease						
Diabetes	32 854 (29.31)	8663 (30.91)	8424 (30.06)	8126 (29.00)	7641 (27.27)	<.001
Glomerulonephritis	12 482 (11.13)	3294 (11.75)	3050 (10.88)	3097 (11.05)	3041 (10.85)	
Hypertension	30 562 (27.26)	5616 (20.04)	7549 (26.94)	8245 (29.42)	9152 (32.66)	
Polycystic kidney disease	9346 (8.34)	2756 (9.83)	2385 (8.51)	2261 (8.07)	1944 (6.94)	
Other	26 283 (23.45)	7546 (26.93)	6483 (23.13)	6162 (21.99)	6092 (21.74)	
Missing	571 (0.51)	150 (0.54)	133 (0.47)	134 (0.48)	154 (0.55)	
Diabetes						
Yes	41 353 (36.89)	10 497 (37.46)	10 559 (37.68)	10 420 (37.18)	9877 (35.24)	<.001
No	69 850 (62.31)	17 425 (62.18)	17 377 (62.01)	17 405 (62.11)	17 643 (62.96)	
Missing	895 (1.16)	103 (0.51)	88 (0.53)	200 (1.48)	504 (2.09)	
Chronic obstructive pulmonary disease						
Yes	1469 (1.31)	434 (1.55)	390 (1.39)	379 (1.35)	266 (0.95)	<.001
No	101 205 (90.28)	24 103 (86.01)	25 103 (89.58)	25 902 (92.42)	26 097 (93.12)	
Missing	9424 (8.41)	3488 (12.45)	2531 (9.03)	1744 (6.22)	1661 (5.93)	
**Donor characteristic**						
Age, y						
<18	11 266 (10.05)	2697 (10.05)	2848 (10.05)	2812 (10.05)	2909 (10.05)	<.001
18-50	69 483 (61.98)	17 690 (63.12)	17 581 (62.74)	17 453 (62.28)	16 759 (59.80)	
>50	31 349 (27.97)	7638 (27.25)	7595 (27.10)	7760 (27.69)	8356 (29.82)	
KDPI, %						
<0.25	34 606 (30.87)	9430 (33.65)	8669 (30.93)	8437 (30.11)	8070 (28.80)	<.001
0.25-0.50	31 471 (28.07)	8022 (28.62)	8097 (28.89)	7890 (28.15)	7462 (26.63)	
0.51-0.84	36 289 (32.37)	8661 (30.9)	9075 (32.38)	9218 (32.89)	9335 (33.31)	
≥0.85	9051 (8.07)	1811 (6.46)	2061 (7.35)	2302 (8.21)	2877 (10.27)	
Missing	681 (0.61)	101 (0.36)	122 (0.44)	178 (0.64)	280 (1.00)	
Sex						
Women	44 814 (39.98)	11 073 (39.51)	11 235 (40.09)	11 277 (40.24)	11 229 (40.07)	.31
Men	67 284 (60.02)	16 952 (60.49)	16 789 (59.91)	16 748 (59.76)	16 795 (59.93)	
Race and ethnicity						
Black	15 550 (13.87)	2814 (10.04)	4028 (14.37)	4317 (15.4)	4391 (15.67)	
Hispanic	15 189 (13.55)	3836 (13.69)	3711 (13.24)	3226 (11.51)	4416 (15.76)	
White	77 508 (69.14)	20 268 (72.32)	19 373 (69.13)	19 684 (70.24)	18 183 (64.88)	
Other^f^	3851 (3.44)	1107 (3.95)	912 (3.25)	798 (2.85)	1034 (3.69)	
BMI						
<18.5	6616 (5.90)	1572 (5.61)	1698 (6.06)	1571 (5.61)	1775 (6.33)	<.001
18.5-24.9	38 504 (34.35)	9482 (33.83)	9462 (33.76)	9704 (34.63)	9856 (35.17)	
25-29.9	34 735 (30.99)	8807 (31.43)	8568 (30.57)	8636 (30.82)	8724 (31.13)	
≥30	32 082 (28.62)	8128 (29)	8263 (29.49)	8081 (28.83)	7610 (27.16)	
Missing	161 (0.14)	36 (0.13)	33 (0.12)	33 (0.12)	59 (0.21)	
Hypertension						
No	80 582 (71.89)	20 594 (73.48)	20 161 (71.94)	19 967 (71.25)	19 860 (70.87)	<.001
Yes	31 516 (28.11)	7431 (26.52)	7863 (28.06)	8058 (28.75)	8164 (29.13)	
**Transplant factor**						
Donor-recipient CMV seropairing						
D-, R-	11 032 (9.84)	2813 (10.04)	2713 (9.68)	2931 (10.46)	2575 (9.19)	<.001
R+	61 398 (54.77)	12 174 (43.44)	13 872 (49.5)	16 967 (60.54)	18 385 (65.60)	
D+, R-	16 592 (14.8)	3931 (14.03)	4240 (15.13)	4318 (15.41)	4103 (14.64)	
Missing	23 076 (20.59)	9107 (32.5)	7199 (25.69)	3809 (13.59)	2961 (10.57)	
HLA mismatch						
0	8128 (7.25)	1912 (6.82)	1776 (6.34)	1892 (6.75)	2548 (9.09)	<.001
1-2	6024 (5.37)	1574 (5.62)	1487 (5.31)	1403 (5.01)	1560 (5.57)	
3-6	97 067 (86.59)	24360 (86.92)	24 603 (87.79)	24 517 (87.48)	23 587 (84.17)	
Missing	879 (0.78)	179 (0.64)	158 (0.56)	213 (0.76)	329 (1.17)	
Cold ischemia, h						
<12	32 542 (29.03)	9132 (32.59)	8346 (29.78)	8294 (29.60)	6770 (24.16)	<.001
12-24	54 649 (48.75)	13 514 (48.22)	13 497 (48.16)	13 692 (48.86)	13 946 (49.76)	
>24	21 068 (18.79)	4752 (16.96)	5265 (18.79)	5198 (18.55)	5853 (20.89)	
Missing	3839 (3.42)	627 (2.24)	916 (3.27)	841 (3.00)	1455 (5.19)	
**Contextual characteristic**						
Area deprivation index, median (IQR), %	53.7 (31.12-72.32)	53.27 (34.29-70.38)	55.15 (33.04-72.33)	56.93 (33.95-75.15)	47.01 (24.21-71.05)	<.001
High school graduates, median (IQR), %	60.05 (51.80-65.82)	62.24 (55.43-67.40)	60.34 (52.45-65.82)	59.55 (51.11-65.71)	57.2 (48.86-63.87)	<.001
Household income, median (IQR), $	51 596 (40 731-66 575)	53 006 (43 655-66 637)	52 122 (41 203-67 840)	50 750 (39 249-66 655)	50 243 (38 450-65 088)	<.001
Income below poverty level, median (IQR), %	15.67 (10.03-23.02)	13.9 (9.30-19.62)	15.32 (9.65-22.14)	16.21 (10.15-24.35)	18.37 (11.43-26.45)	<.001
Unemployment, median (IQR), %	8.70 (6.56-11.78)	7.85 (5.92-10.11)	8.47 (6.39-11.14)	8.87 (6.61-12.10)	10.03 (7.43-13.67)	<.001
Population concentration, median (IQR), individuals/m^2^	0.00076 (0.00013-0.00186)	0.00018 (0.000037-0.00082)	0.00063 (0.00013-0.00155)	0.00099 (0.00026-0.00196)	0.00160 (0.00062-0.00363)	<.001
**Outcome**						
Follow-up, median (IQR), y	5.95 (3.85-8.90)	5.02 (3.70-7.23)	5.48 (3.82-7.89)	6.61 (3.98-9.04)	7.33 (3.96-10.92)	<.001
Acute kidney rejection						
Yes	7793 (6.95)	1762 (6.29)	1821 (6.50)	2105 (7.51)	2105 (7.51)	<.001
No	79 048 (70.52)	20 382 (72.73)	20 089 (71.68)	19 536 (69.71)	19 041 (67.95)	
Missing	25 257 (22.53)	5881 (20.98)	6114 (21.82)	6384 (22.78)	6878 (24.54)	
Death-censored graft failure						
Yes	18 652 (16.64)	2974 (10.61)	3831 (13.67)	5104 (18.21)	6743 (24.06)	<.001
No	93 446 (83.36)	25 051 (89.39)	24 193 (86.33)	22 921 (81.79)	21 281 (75.94)	
All-cause death						
Yes	27 826 (24.82)	5047 (18.01)	5670 (20.23)	7336 (26.18)	9773 (34.87)	<.001
No	84 272 (75.18)	22 978 (81.99)	22 354 (79.77)	20 689 (73.82)	18 251 (65.13)	

Abbreviations: BMI, body mass index (calculated as weight in kilograms divided by height in meters squared); CMV, cytomegalovirus; HLA, human leukocyte antigen; KDPI, kidney donor profile index; PM_2.5_, fine particulate matter air pollution.

^a^ Patients are stratified by annual mean PM_2.5_ concentration in the year before kidney transplant (ie, baseline PM_2.5_ level) for patients receiving kidney transplant from 2004 to 2016.

^b^ PM_2.5_ level = 1.2 μg/m^3^ to less than 8.3 μg/m^3^.

^c^ PM_2.5_ level = 8.3 μg/m^3^ to less than 9.8 μg/m^3^.

^d^ PM_2.5_ level = 9.8 μg/m^3^ to less than 11.9 μg/m^3^.

^e^ PM_2.5_ level = 11.9 μg/m^3^ to less than 22.4 μg/m^3^.

^f^ The other race and ethnicity group includes Asian individuals, American Indian or Alaska Native individuals, Native Hawaiian or other Pacific Islander individuals, and individuals of multiple races or ethnicities.

To plot the exposure-response function for death-censored graft failure and all-cause death, cubic spline analyses were first performed in multivariable Cox models with knots placed at baseline PM_2.5_ level quartiles,^39^ and the statistical significance of spline terms was assessed for nonlinearity of spline terms. When there was no evidence of deviation from linearity, patients residing in areas with the lowest 1% (ie, 3.7 μg/m^3^) and the highest 1% (ie, 16.9 μg/m^3^) baseline PM_2.5_ levels were excluded from the analytic cohort, and multivariable-adjusted hazard ratios using the aforementioned Cox models were estimated using 3.7 μg/m^3^ as the reference level. These multivariable-adjusted hazard ratios (HRs) were plotted against the baseline PM_2.5_ level from 3.7 μg/m^3^ to 16.9 μg/m^3^ with the background of the histogram distribution of baseline PM_2.5_ level for the included analytic cohort.^11^

Geographic distribution of the estimated national burden of graft failure associated with PM_2.5_ levels greater than the Environmental Protection Agency (EPA) recommended PM_2.5_ concentration of 12 μg/m^3^ was plotted using the population attributable fraction (PAF) multiplied by incidence of graft failure per 100 000 patients with KTs per year from 2004 to 2016. PAF was the proportional reduction of the condition in the KT population that would occur if exposure to PM_2.5_ was decreased to 12 μg/m^3^. PAF was computed using piecewise constant hazard models for graft failure incidence.^11,40,41^

All tests were 2-sided, and results were considered statistically significant at α = .05 or when 95% CIs did not cross 1 for odds ratios and hazard ratios. All statistical analyses were performed using SAS statistical software version 9.4 (SAS Institute) and R statistical software version 4.0.2 (R Project for Statistical Computing). Zip code–level PM_2.5_ concentration was computed using ArcGIS Pro software version 2.7.0 (Esri). Data were analyzed from April 2020 through July 2021.

To account for variations in city characteristics that could confound the association, sensitivity analyses for all 3 outcomes were conducted using multilevel models and adapting city-adjusted and within-city models. These models were detailed elsewhere.^5,11^ Cities were defined as core-based (including metropolitan and micropolitan) statistical areas, which were obtained from the US Census Bureau^33^ and linked to patient residential zip codes. Additionally, for the acute kidney rejection outcome, multivariable logistic regression was performed adjusting for city clustering.

## Results

Among 214 317 patients who received KTs from 2004 to 2016 in the United States (eFigure in the Supplement), we excluded 9338 patients aged younger than 18 years at KT, 71 536 patients receiving kidneys from living donors, 14 650 patients with a previous KT, 6573 patients with missing residential zip codes, and 122 patients with missing data on at least 1 continuous variable. The resulting analytic cohort included 112 098 patients with KTs. The median (IQR) follow-up was 6.0 (3.9-8.9) years; 70 522 individuals (62.9%) were older than age 50 years at the time of KT and 68 117 (60.8%) were men (Table).

Most patients were White (48 581 patients [43.3%]), while 37 265 patients (33.2%) were Black, 17 047 patients (15.2%) were Hispanic, and 9205 patients (8.2%) were of other race or ethnicity. Most patients had obesity or overweight BMI and were on dialysis more than 24 months before KT. Most patients received kidneys from donors who were aged 18 to 50 years (69 483 patients [62.0%]), men (67 284 patients [60.0%]), and White (77 508 individuals [69.1%]), while 15 550 patients had donors who were Black (14.0%), 15 189 patients had donors who were Hispanic (14.0%), and 3851 patients had donors who were of other race or ethnicity (3.4%). Most patients received kidneys from donors with a BMI from 18.5 to 24.9 (38 504 patients [34.4%]) or 25.0 to 29.9 (34 735 patients [30.99%]). Among transplant factors, most patients had a level of HLA mismatch of 3 to 6 hours and 12 to 24 hours of cold ischemia time. The median (IQR) ADI of patient residential zip code areas was 53.7 (31.1-72.3), and the median (IQR) population density was 0.00076 (0.00013-0.00186) individuals per square meter.

The median (IQR) baseline PM_2.5_ level was 9.8 (8.3-11.9) μg/m^3^. Baseline PM_2.5_ concentration ranged from 1.2 μg/m^3^ to less than 8.3 μg/m^3^ among 28 025 patients in the first quartile, from 8.3 μg/m^3 ^to less than 9.8 μg/m^3^ among 28 024 patients in the second quartile, from 9.8μg/m^3^ to less than 11.9 μg/m^3^ among 28 025 patients in the third quartile, and from 11.9 μg/m^3^to less than 22.4 μg/m^3^ among 28 024 patients in the fourth quartile. All recipient demographics, donor characteristics, transplant factors, and contextual factors were statistically significantly different across PM_2.5_ level quartiles except for sex for recipients and donors. Factors that decreased with PM_2.5_ level quartiles included proportion of recipients aged older than 50 years (quartile 1: 18 413 patients [65.7%]; quartile 2: 17 664 patients [63.0%]; quartile 3: 17 403 patients [62.1%]; quartile 4: 17 042 patients [60.8%]; P < .001) and the proportion who were White (quartile 1: 15 765 White individuals [56.3%]; 5148 Black individuals [18.4%]; 4323 Hispanic individuals [15.4%]; 2789 individuals with other race or ethnicity [10.0%]; quartile 2: 12 099 White individuals [43.2%]; 9572 Black individuals [34.2%]; 4130 Hispanic individuals [14.7%]; 2223 individuals with other race or ethnicity [7.9%]; quartile 3: 11 255 White individuals [40.2%]; 11 202 Black individuals [40.0%]; 3537 Hispanic individuals [12.6%]; 2031 individuals with other race or ethnicity [7.3%]; quartile 4: 9462 White individuals [33.8%]; 11 343 Black individuals [40.5%]; 5057 Hispanic individuals [18.1%]; 2162 individuals with other race or ethnicity [7.7%]; *P* < .001) (Table).

**Figure 1.**
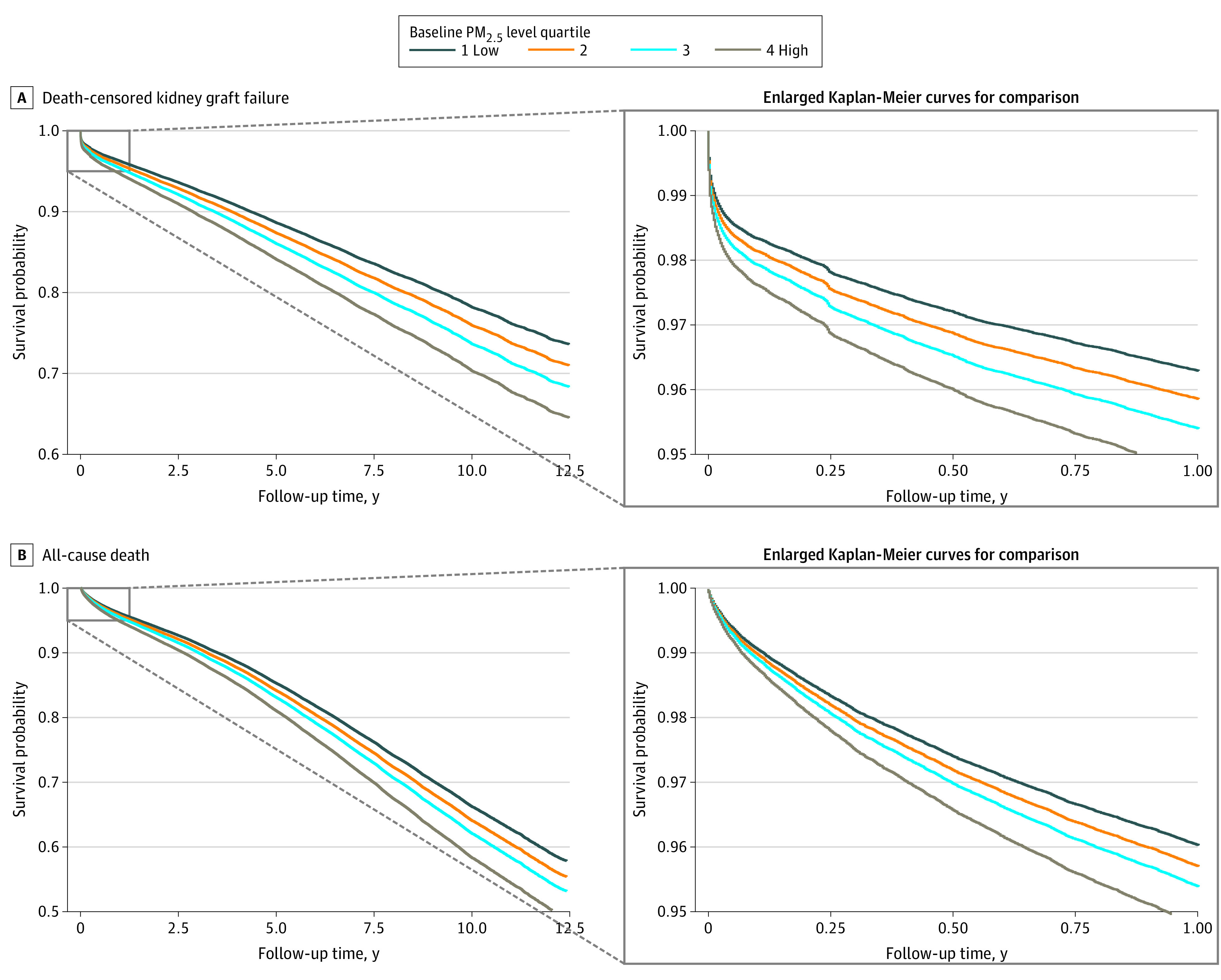
Adjusted Survival Curves by PM_2.5_ Level Quartile Survival curves were adjusted for recipients' age, sex, race and ethnicity, dialysis status and duration, and area deprivation index. PM_2.5_ indicates fine particulate matter air pollution.

Other factors that decreased by quartile included diabetes as a comorbidity, hypertension as etiology of kidney disease, PRA of 0%, and public insurance, as well as proportion of KTs with cold ischemia time less than 12 hours. Patients receiving KTs in earlier years were more likely to be in the fourth quartile, and patients receiving KTs in more recent years were more likely to be in the first quartile, as suggested by the increased follow-up time with quartiles in Table. Increased baseline PM_2.5_ levels by quartile were associated with increases in acute kidney rejection (quartile 1: 1762 patients [6.3%]; quartile 3: 1821 patients [6.5%]; quartile 3: 2105 patients [7.5%]; quartile 4: 2105 patients [7.5%]; *P* < .001), death-censored graft failure (quartile 1: 2974 patients [10.6%]; quartile 2: 3831 patients [13.7%]; quartile 3: 5104 patients [18.2]; quartile 4: 6743 patients [24.1%]; *P* < .001), and all-cause death (quartile 1: 5047 patients [18.0%]; quartile 2: 5670 patients [20.2%]; quartile 3: 7336 patients [26.2%]; quartile 4: 9773 patients [34.9%]; *P* < .001) (Table). The adjusted survival curves on death-censored graft failure and all-cause death, stratified by baseline PM_2.5_ level quartiles (Figure 1A-B) demonstrated a similar pattern.

**Figure 2.**
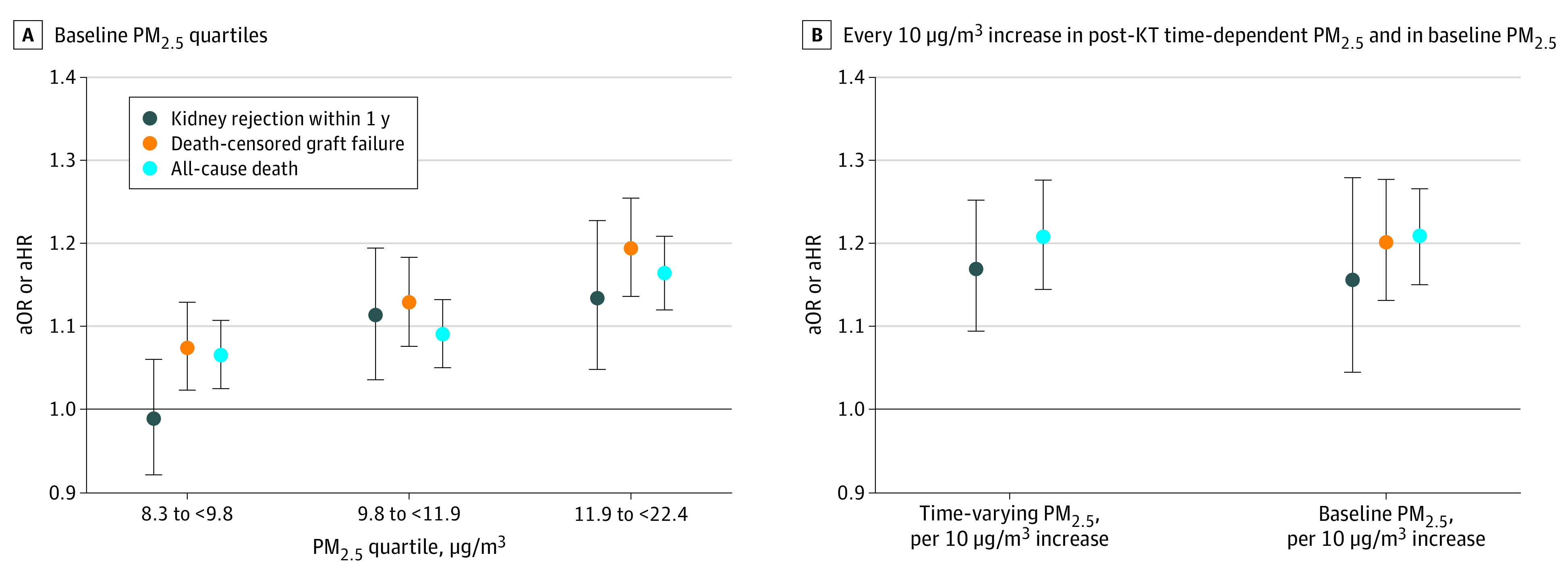
Odds of Acute Kidney Rejection and Risk of Death-Censored Graft Failure and All-Cause Death All models were adjusted for recipient characteristics, donor characteristics, transplant factors, and contextual factors. Outcomes are reported as adjusted odds ratios (aORs) for acute kidney rejection and adjusted hazard ratios (aHRs) for death-censored graft failure and all-cause death by baseline fine particulate matter (PM_2.5_) air pollution level quartiles (reference: first quartile, PM_2.5_ level = 1.2 μg/m^3^-8.3 μg/m^3^) or every 10 μg/m^3^ increase in post-KT time-dependent PM_2.5_ level and baseline PM_2.5_ level.

**Figure 3.**
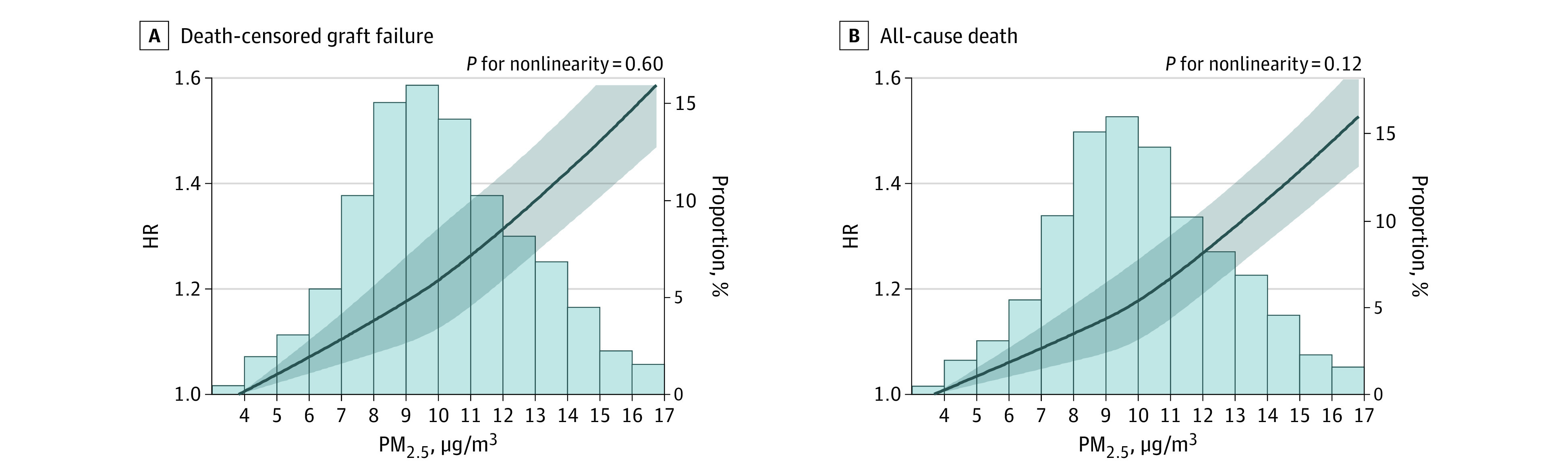
Risk of Outcomes With PM_2.5_ Level Distribution in Background All models were adjusted for recipient characteristics, donor characteristics, transplant factors, and contextual factors. The reference level was PM_2.5_ = 3.7 μg/m^3^. HR indicates hazard ratio; PM_2.5_, fine particulate matter air pollution. Shaded areas indicate 95% CIs; histograms, distribution of PM_2.5_ level.

 In multivariable analyses (Figure 2), compared with quartile 1 of baseline PM_2.5_ level (Figure 2A), the odds of acute kidney rejection did not increase statistically significantly for quartile 2 (adjusted odds ratio [aOR], 0.99; 95% CI, 0.92-1.06) but did for quartile 3 (aOR, 1.11; 95% CI, 1.04-1.20) and quartile 4 (aOR, 1.13; 95% CI, 1.05-1.23). For the exposure response function, cubic spline analyses suggested no evidence of nonlinear association between PM_2.5_ concentration and risks for death-censored graft failure or all-cause death. These analyses are presented in Figure 3 with the background of the histogram distribution of the baseline PM_2.5_ level.

Risks for adverse KT outcomes increased with levels of post-KT time-dependent PM_2.5_ (death-censored graft failure: adjusted hazard ratio [aHR] per 10 μg/m^3^ increase, 1.17; 95% CI, 1.09-1.25; all-cause death: aHR per 10 μg/m^3^ increase, 1.21; 95% CI, 1.14-1.28) (Figure 2B left). Using baseline PM_2.5_ level by quartile as exposure, we found that increased baseline PM_2.5_ level, compared with baseline PM_2.5_ level at quartile 1, was associated with increased risk for death-censored graft failure (quartile 2: aHR, 1.08; 95% CI, 1.02-1.13; quartile 3: aHR, 1.13; 95% CI, 1.08-1.18; quartile 4: aHR, 1.19; 95% CI, 1.14-1.26) and all-cause death (quartile 2: aHR, 1.07; 95% CI, 1.03-1.11; quartile 3: aHR, 1.09; 95% CI, 1.05-1.13; quartile 4: aHR, 1.16; 95%, CI, 1.12-1.21). Using continuous baseline PM_2.5_ exposure (Figure 2B, right), increased PM_2.5_ levels were associated with increased odds for the 3 KT outcomes per 10 μg/m^3^ increase in PM_2.5_ concentration (rejection: aOR, 1.16; 95% CI, 1.04-1.28; graft failure: aHR, 1.20; 95% CI, 1.13-1.28; death: aHR, 1.21; 95% CI, 1.15-1.27).

**Figure 4.**
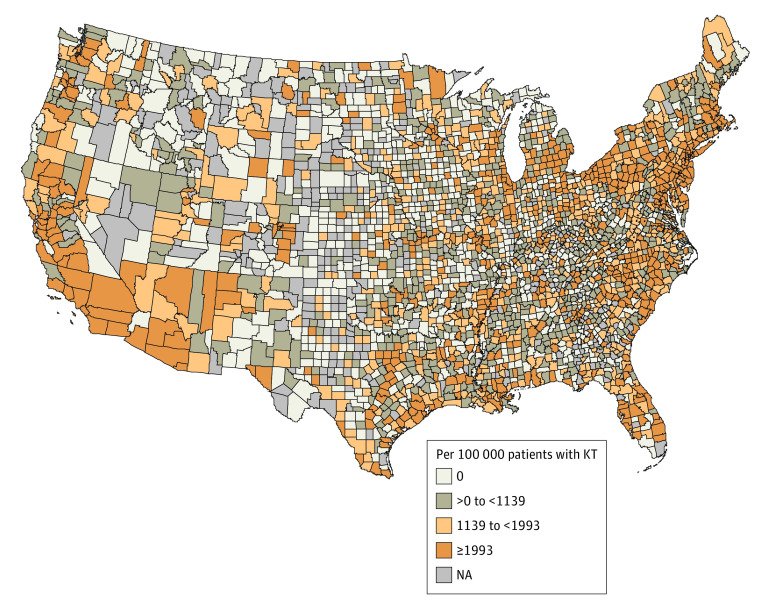
Geographic Distribution of National Burden of Graft Failure Graft failure associated with fine particulate matter air pollution (PM_2.5_) levels above the Environmental Protection Agency recommended concentration of 12 μg/m^3^ in the United States per year is shown per 100 000 patients with kidney transplants (KTs) from 2004 to 2016.

The PAF for graft failure if exposure to PM_2.5_ was reduced to the EPA recommended level of 12 μg/m^3^ was 3.99% (95% CI 3.32%-4.65%). The national burden of graft failure associated with increased levels of PM_2.5_ over 12 μg/m^3^ was estimated to be 57 failures (95% uncertainty interval, 48 failures-67 failures) among 8623 patients with KTs per year from 2004 to 2016. The map illustrating the geographic distribution of the burden of graft failure (per 100 000 patients with KTs) associated with increased levels of PM_2.5_ over 12 μg/m^3^ is presented in Figure 4. The burden increased with the darkness of the color, and the areas with gray color indicate that no patients in the analytic cohort resided in those areas at the time of their KTs. In sensitivity analyses adjusting for variations in city characteristics, we found that, compared with quartile 1 of the baseline PM_2.5_ level, increased baseline PM_2.5_ level quartiles were associated with increased risk of acute kidney rejection, graft failure, and all-cause death (eTable in the Supplement).

## Discussion

This cohort study is one of the first studies, to our knowledge, to assess the association of ambient fine particulate matter air pollution with outcomes among recipients of KTs. Using annual mean PM_2.5_ concentration during post-KT follow-up or in the year before KT (by quartile or quantity), our study consistently found that PM_2.5_ concentration was an independent risk factor associated with acute rejection, death-censored graft failure, and all-cause mortality among recipients of KTs. These results were robust when different statistical models (with or without adjustment for city variations) were used. We also found linear exposure response associations between baseline PM_2.5_ concentration and risks for death-censored graft failure and all-cause death. These findings suggest that consistent exposure to fine particulate matter air pollution is associated with increased risk of worse transplant outcomes among recipients of KTs, including kidney rejection, kidney graft failure, and all-cause death.

The geographic distribution of the burden of graft failure associated with increased levels of PM_2.5_ over 12 μg/m^3^ suggests that the highest burden was concentrated in areas with high population density and a high degree of air pollution, such as the Southwest and East North Central regions. The map showing areas with increased burden is consistent with that in Goodkind et al,^42^ in which the authors illustrated the estimated monetary marginal damages at every emission source location on a map.^42^

One highlight of this study is the finding that increased PM_2.5_ concentration was associated with increased risk of kidney graft failure. This finding is consistent with those in previous reports finding increased risks for CKD and ESRD among individuals with native kidneys.^11,13,43^ Additionally, using multiple definitions of exposures, Bowe et al^11^ found an association between exposure to PM_2.5_ and risk for incident CKD and ESRD in a cohort of US veterans. In an earlier study, Mehta et al^13^ found that 1-year exposure to increased PM_2.5_ concentration was associated with an annual decrease in kidney function. Globally, it was estimated that PM_2.5_ concentration is associated with 3.3 million cases of incident CKD and 122.4 million cases of prevalent CKD.^43^ However, our finding is not supported by the finding in a Feng et al,^44^ which found that risk of death-censored graft failure was increased with increased PM_2.5_ concentrations, although this change was not statistically significant. This deviation could be associated with a shorter follow-up time (ie, 2.5-9.5 years in Feng et al^44^ vs 2-15 years for analyses using time-dependent exposure and 4.25-17.25 years for analyses using baseline exposure in this study).

Kidney graft rejection is a major risk factor associated with graft loss.^45,46,47^ We found a 13% increase in odds of rejection within the first year of KT among recipients residing in areas with the fourth quartile of baseline PM_2.5_ levels, compared with the first quartile. This finding is consistent with that in Feng et al^44^ and suggests an alloimmune etiology as a possible pathway for rejection that may be associated with increased risk of graft loss. The exact mechanism of increased risk of rejection with PM_2.5_ has not yet been elucidated. We hypothesize that this could be associated with increased systemic inflammation and activation of the innate and adaptive immune systems. This hypothesis is based on a growing body of literature suggesting that the organic compounds, free radicals, and transition metals contained in PM_2.5_ are associated with increased oxidative stress, as well as the gene and protein expression of proinflammatory mediators, such as tumor necrosis growth factor α, monocyte chemoattractant protein 1, macrophage inflammatory protein 2, interleukin 6, interleukin 1β, and interleukin 8.^15,48^ Studies have also found that PM_2.5_ is associated with increased expression of adhesion molecules like vascular cellular adhesion molecule 1 and the adhesion of monocyte cells to endothelial cells.^49,50^ In lung transplant, aryl hydrocarbon receptor is considered as a pathway to changing naive T cells to inflammatory T helper 17 cells and promoting chronic inflammation and chronic rejection.^51^

Our study found a 21% increase in mortality risk among individuals with KTs for every 10 ug/m^3^ increase in PM_2.5_ level, similar to the finding of Feng et al (15% increase per 10 ug/m^3^ increase in PM_2.5_ level).^44^ Prior studies among recipients of KTs have found increased risk of cardiovascular mortality from exposure to air pollutants.^25,26^ The increased mortality risk has also been reported among individuals with heart transplants: 26% to 43% increases in mortality risk per 10 ug/m^3^ increase in time-dependent PM_2.5_ concentration.^24^ In patients with lung transplants, the increase in mortality risk was not statistically significant, as reported by Bhinder and colleagues,^52^ possibly associated with the exposure definition (ie, mean annual PM_2.5_ concentration from 1996-2010) and a smaller sample size (ie, approximately 400 individuals). As for mortality among patients with CKD, it was estimated at 211 019 deaths associated with CKD associated with PM_2.5_ exposure globally.^43^ However, similar to the situation in the general nontransplant population, it is likely that most of these deaths are associated with detrimental cardiovascular outcomes of PM_2.5_ exposure.^14,53^

### Strengths and Limitations

This study has several strengths. First, to our knowledge, it is one of the first studies on the association of PM_2.5_ levels with acute rejection, graft failure, and death in a large national cohort of individuals with KTs. Second, our analyses benefited from merges of multiple databases to comprehensively account for potential confounding, including patient, donor, and transplant factors, as well as contextual characteristics (eg, ADI and population density). Third, multiple exposure definitions were used to ensure the robustness of study findings. Fourth, the robustness of the findings was strengthened by sensitivity analyses adjusting for city variations. We note that PM_2.5_ concentration generally decreased over time. As a consequence, using time-dependent exposure is particularly important to capture the association of decreasing PM_2.5_ levels over time with transplant outcomes. Furthermore, this trend was associated with changes in included recipient and donor characteristics, transplant factors, and contextual factors, as well as transplant outcomes when recipients were grouped by baseline PM_2.5_ level quartiles, as presented in the Table. Patients receiving KTs in earlier years were more likely to be in the fourth quartile, while patients receiving KTs in more recent years were more likely to be in the first quartile. To account for these differences, we used multivariable time-to-event analyses with all relevant factors (including year of KT) included as covariates. We then chose to report the results using time-dependent exposure as the main findings, supplemented with results using baseline exposures.

Nonetheless, this study has several limitations that should be noted. First, like most retrospective studies, the results rely on the accuracy of the recorded data from multiple databases. Second, although time-dependent analyses allowed for capturing the exposure after KT, the most updated annual mean PM_2.5_ concentration was available up to 2018 at the time of the study, limiting follow-up time. Nonetheless, we were able to include a follow-up of 2 to 15 years. Third, the most up-to-date residential zip codes for recipients of KTs were recorded at the time of KT. Time-dependent analyses may be biased if the patients moved to another zip code area after KT. However, this bias may be decreased if patients who moved after KT were not systematically more likely to move to areas with higher or lower levels of PM_2.5_ compared with their area of residence at KT. Fourth, composition and toxic content of PM_2.5_ may change over time and by geography; consequently, use of PM_2.5_ level alone may underestimate risk.^54^ Fifth, indoor air pollution was not accounted for in this study. Sixth, although we controlled for as many covariates as possible and adjusted for city variations, residual confounding may remain, which could bias the estimated association.

## Conclusions

To our knowledge, this is one of the first studies in a national cohort of recipients of KTs that found that increased levels of PM_2.5_ were independently associated with increased risk of acute rejection, graft loss, and death. Our findings suggest that efforts toward cleaner air may be associated with decreased burden of adverse outcomes after KT. In clinical practice, suggesting that recipients of KTs reside in areas with lower levels of PM_2.5_ concentration may be associated with improved transplant outcomes.

## Notes

Supplement. **eFigure.** Patient Attrition Diagram **eTable.** Risk of Death-Censored Graft Failure and All-Cause Death and Odds of 1-Year Kidney Rejection by Quartile
