# Supplementary material for: Association of Ambient Fine Particulate Matter Air Pollution With Kidney Transplant Outcomes
Source: JAMA Netw Open. 2021 Oct 7;4(10):e2128190. doi: 10.1001/jamanetworkopen.2021.28190 (PMC8498852; doi:10.1001/jamanetworkopen.2021.28190)
Supplement: Supplement. — eFigure. Patient Attrition Diagram eTable. Risk of Death-Censored Graft Failure and All-Cause Death and Odds of 1-Year Kidney Rejection by Quartile [file jamanetwopen-e2128190-s001.pdf]

Supplemental Online Content

Chang SH, Merzkani M, Murad H, et al. Association of ambient fine particulate matter air pollution with kidney transplant outcomes. *JAMA Netw Open*. 2021;4(10):e2128190.
doi:10.1001/jamanetworkopen.2021.28190

eFigure. Patient Attrition Diagram

eTable. Risk of Death-Censored Graft Failure and All-Cause Death and Odds of 1-Year Kidney Rejection by Quartile

This supplemental material has been provided by the authors to give readers additional information about their work.

© 2021 Chang SH et al. *JAMA Network Open*.

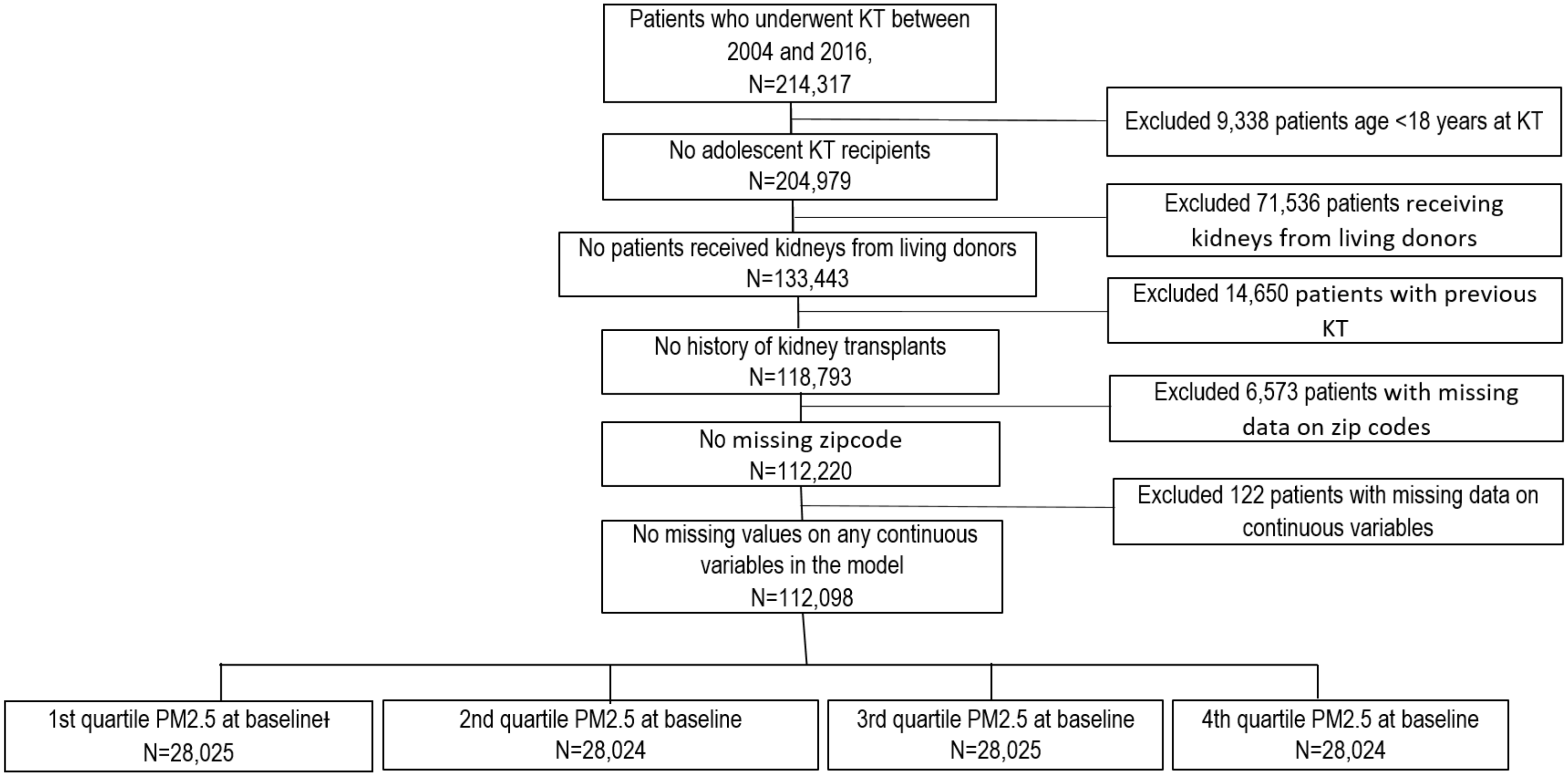

eFigure. Patient Attrition Diagram

© 2021 Chang SH et al. *JAMA Network Open.*

eTable. Risk of Death-Censored Graft Failure and All-Cause Death and Odds of 1-Year Kidney Rejection by Quartile

Model		Quartiles (Ref: Quartile 1)	1-year kidney rejection, aOR (95% CI)	Graft failure aHR (95% CI)*	All-cause death aHR (95% CI)*
Logistic regression adjusting for city clustering		Quartile 2	0.99 (0.92, 1.06)	-- (--, --)	-- (--, --)
		Quartile 3	1.06 (0.96, 1.16)	-- (--, --)	-- (--, --)
		Quartile 4	1.08 (0.96, 1.21)	-- (--, --)	-- (--, --)
City-adjusted model		Quartile 2	0.98 (0.90, 1.07)	1.07 (1.01, 1.13)	1.07 (1.02, 1.12)
		Quartile 3	1.06 (0.96, 1.17)	1.13 (1.06, 1.20)	1.10 (1.05, 1.16)
		Quartile 4	1.11 (0.99, 1.25)	1.22 (1.14, 1.31)	1.18 (1.11, 1.25)
Within city model	Between-city	Quartile 2	1.12 (1.04, 1.19)	1.08 (1.04, 1.13)	1.04 (1.00, 1.07)
		Quartile 3	1.07 (0.99, 1.15)	1.13 (1.08, 1.19)	1.07 (1.03, 1.11)
		Quartile 4	1.17 (1.09, 1.26)	1.10 (1.05, 1.15)	1.07 (1.03, 1.11)
	Within-city	Quartile 2	1.05 (0.98, 1.13)	1.00 (0.95, 1.05)	0.99 (0.95, 1.03)
		Quartile 3	1.09 (1.01, 1.17)	1.09 (1.03, 1.15)	1.05 (1.00, 1.09)
		Quartile 4	1.07 (0.99, 1.17)	1.13 (1.07, 1.19)	1.11 (1.06, 1.16)

All models adjusted for all recipient characteristics, donor characteristics, transplant factors, contextual factors, and city clustering. Outcomes are reported as adjsted hazard ratios for graft failure and all-cause death and odds ratios for 1-year kidney rejection by baseline $PM_{2.5}$ quartile (reference: 1st quartile: $PM_{2.5}$,1.2-<8.3 μg/m3), additionally adjusted for city clustering effect.

*Robust sandwich variance estimators were used.

© 2021 Chang SH et al. *JAMA Network Open.*
